# The composition of polypharmacy: A register-based study of Swedes aged 75 years and older

**DOI:** 10.1371/journal.pone.0194892

**Published:** 2018-03-29

**Authors:** Jonas W. Wastesson, Angel Cedazo Minguez, Johan Fastbom, Silvia Maioli, Kristina Johnell

**Affiliations:** 1 Aging Research Center, Department of Neurobiology, Care Sciences and Society, Karolinska Institutet & Stockholm University, Stockholm, Sweden; 2 Department of Neurobiology, Care Sciences and Society, Center for Alzheimer Research, Division for Neurogeriatrics, Karolinska Institutet, Huddinge, Stockholm, Sweden; Cardiff University, UNITED KINGDOM

## Abstract

**Background:**

Polypharmacy is common among older adults. However, little is known about the composition of polypharmacy: which are the most frequently used drugs, and how much do these drugs contribute to the overall prevalence of polypharmacy.

**Methods:**

A total of 822,619 Swedes aged ≥75 years was identified from the Total Population Register. Through record-linkage with the Swedish Prescribed Drug Register and the Social Services Register we could analyze concurrent drug use in the entire population (both individuals living in the community and institution) on the 31 December 2013.

**Results:**

The prevalence of polypharmacy (≥5 drugs) was 45%. The most frequently used drugs were cardiovascular drugs, analgesics, and psychotropics. By excluding the ten most frequently used drug classes or compounds, the prevalence of polypharmacy was reduced by 69% and 51% respectively. The majority of the users of either one of the 10 most frequently used drugs concurrently used at least 4 other drug classes (66%-85%).

**Conclusion:**

Almost half of the individuals aged ≥75 years are exposed to polypharmacy in Sweden. A handful of drugs make a large contribution to the overall prevalence of polypharmacy and the majority of drugs prescribed to persons aged ≥75 years are used in combination with other drugs. This highlights the high use of drugs, and the need to consider other concurrent drug treatments when prescribing for older adults.

## Introduction

The prevalence of polypharmacy (commonly defined as the use of ≥5 drugs) is increasing among older adults in high income countries [1–3]. In Sweden, the prevalence of polypharmacy was 39% among people aged ≥65 years living in the community and 76% among people living in institutions in 2009 [4]. Polypharmacy increases the risk of drug-drug interactions and adverse drug reactions [1,5,6]. It has also been linked to a number of negative health outcomes, such as falls and hospitalization, but these results are less conclusive [7].

Physiological changes in the aging body, multimorbidity and the use of multiple medications complicates prescribing to older adults [8]. For similar reasons, older adults are rarely included in clinical drug trials [9–11]. However, in the real-world setting older adults are the main users of prescribed drugs and many are both frail and use multiple drugs concomitantly [12]. Few studies have investigated to what magnitude the drugs prescribed to older adults are intended to be used as a part of a polypharmacy regimen.

The drug use among older adults, with or without polypharmacy, tends to be concentrated around a limited number of frequently used drugs classes, specifically cardiovascular drugs, analgesics and psychotropic drugs [1,13–16]. Although some notable differences in prescribing patterns has been reported for people residing in the community compared to persons living in institutions [4,13,17]. To our knowledge, however, no efforts have previously been made to estimate how the most frequently prescribed drugs contribute to the overall prevalence of polypharmacy. More evidence regarding which drugs that frequently contribute to polypharmacy might inform the interventions aimed at reducing polypharmacy in older adults [18–22].

To this end, we aim to investigate: 1) the 10 most frequently used drug compounds and drug classes used by Swedes aged ≥75 years with polypharmacy, with excessive polypharmacy, or living in a nursing home; 2) the reduction in the prevalence of polypharmacy when excluding the top-1 through top-10 most frequently used drug classes and drug compounds; and 3) the number of other drugs used concomitantly with the 10 most frequently used drug compounds and drug classes.

## Methods

All persons aged ≥75 years in Sweden were included in the study population (n = 822,619), based on the Register of the Total Population (alive 31 December 2013). Through record-linkage, we were able to obtain information about living arrangement (community-living vs. institution) from the Swedish Social Services Register (SSSR) and drug use from the Swedish Prescribed Drug Register (SPDR). Individual-level linkage of registers is made possible through the personal identity numbers used in Sweden [23]. This study was approved by the Regional Ethical Review Board in Stockholm (dnr 2013/1941-31/3 and 2015/1319-32). All procedures performed in studies involving human participants were in accordance with the ethical standards of the institutional and/or national research committee and with the 1964 Helsinki declaration and its later amendments or comparable ethical standards. For this type of register study formal consent is not required.

The SPDR is one of the largest pharmacoepidemiological databases in the world and includes all prescribed dispensed drugs in Sweden. From the SPDR, we obtained data about dispensed drugs (dispensing date, the amount of drug dispensed, and the prescribed dosage) from 1 October to 31 December 2013. Based on this three-month period we construct a list of concurrently used drugs on the arbitrarily chosen date of 31 December 2013 (1-day point prevalence). If the same drug was prescribed twice, or at different doses, it was treated as one prescription. For a more detailed description of the methodology, please see Wastesson et al [13]. The drugs were classified according to the Anatomical Therapeutic Chemical (ATC) classification system as recommended by the WHO [24].

Information about living arrangement (community-living vs. institution) was derived from the Swedish Social Services Register (SSSR). This register holds detailed information on publically funded social care for persons aged ≥65 years in Sweden [25]. Other actors than the municipalities finance a negligible proportion of the social care in Sweden. Living arrangement was assessed at the same date as the drug use, i.e. 31 December 2013.

### Measures

The number of used drugs was defined as the number of used original drug compounds (5^th^ level ATC code: chemical substance). The number of used drugs was classified as ‘no polypharmacy’ 0–4 drugs, ‘polypharmacy’ ≥5 drugs, and ‘excessive polypharmacy’ ≥10 drugs.

Living arrangement was classified as living in the community or living in an institution. In Sweden, a needs-assessor decides if a person is eligible for institutional care. Hence, people living in institutions are generally a frail sub-population with high care needs. It has been estimated that two-thirds of them have functional disability, and three out of four have cognitive impairment [26]. Therefore, institutionalization in the Swedish context may be used as a proxy for frailty.

The ten most frequently used drug classes (3^rd^ level ATC code: pharmacological subgroup) and drug compounds (5^th^ level ATC code: chemical substance) were calculated for people with polypharmacy, excessive polypharmacy and people living in institutions.

### Statistical analyses

**Fig 1:**
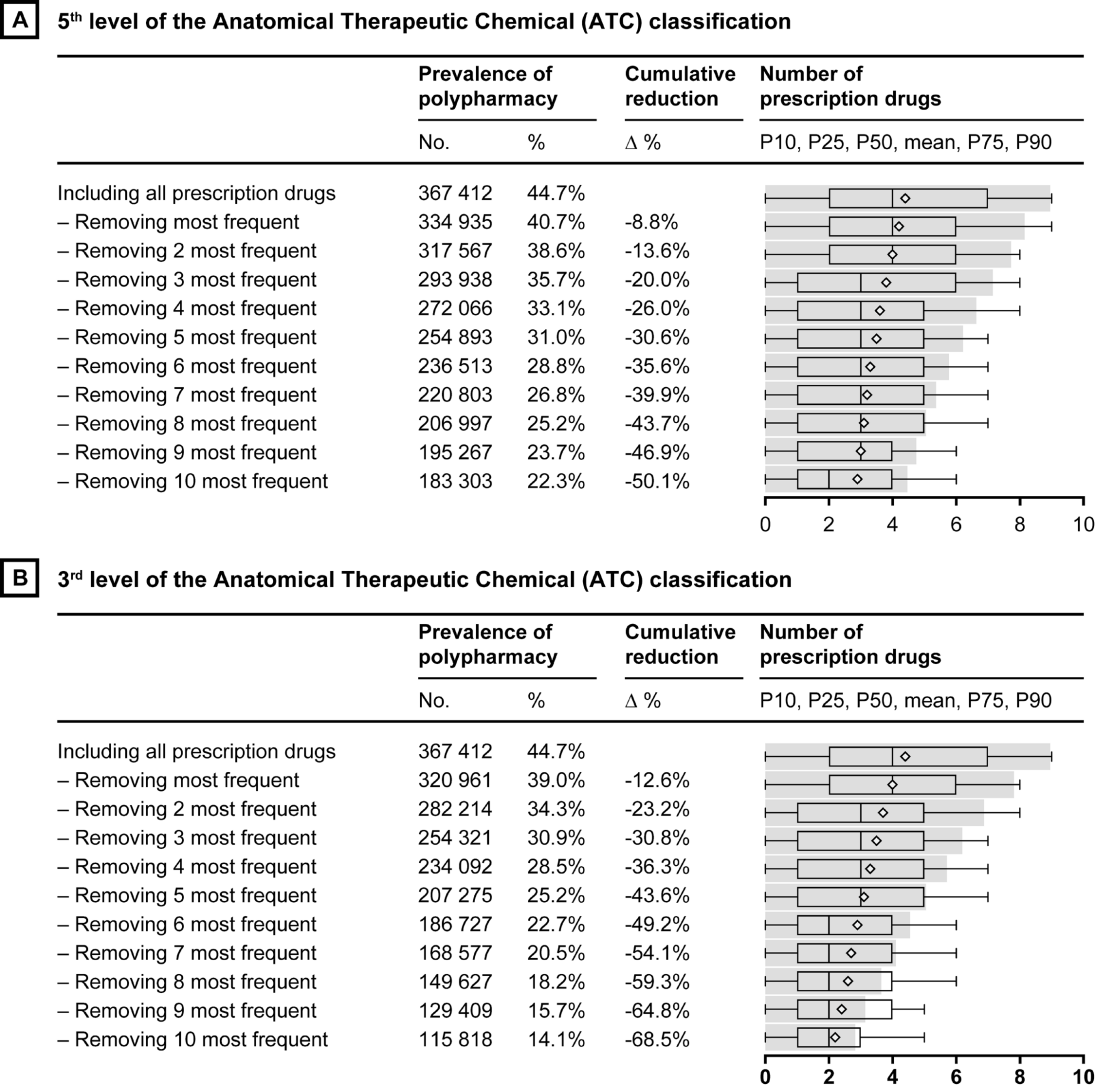
Prevalence of polypharmacy and number of drugs used (mean and median) after exclusion of the top 1 to 10 most frequently used drugs among individuals aged ≥75 years, Sweden 2013.

**Fig 2:**
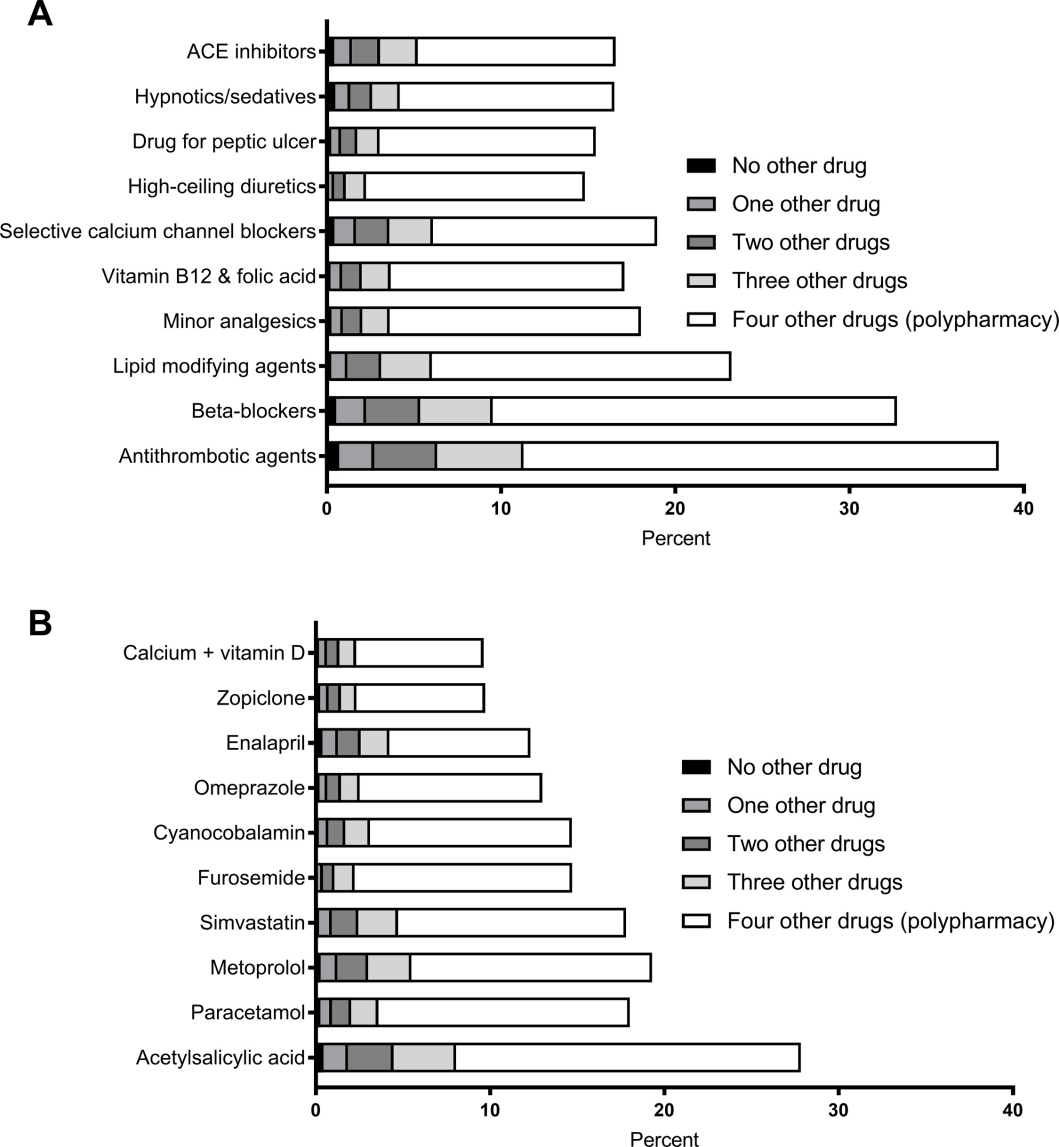
Prevalence and concomitant use of other drugs for A: drug classes (3rd level ATC) and B. drug compounds (5th level ATC), in the total study population (n = 822,619) Sweden 2013.

The prevalence of polypharmacy was calculated for the total study population and for people living in institutions. The ten most frequently used drug classes and drug compounds were calculated for people with polypharmacy, excessive polypharmacy and people living in institutions, respectively. We also calculated the most frequently used drug classes for the population aged 65–74 years (n = 1,051,115) to investigate age differences in commonly used drugs (presented in the Supporting Material, S1 Table). This population was extracted from the same registers, using the same methodology, as for the population aged ≥75 years. We calculated the reduction in the prevalence of polypharmacy, together with changes in the mean and median number of used drugs, when excluding the most frequently used drugs (Fig 1). Last, the number of other drugs used concurrently with the ten most frequently used drug classes and drug compounds by the patient was calculated (Fig 2). STATA 14 (Stata Corp LP, College Station, TX) was used for the analyses and data preparation was done using a customized drug data software (Monitior).

## Results

In the total study population of 822,619 Swedes 75 years and older, 45% (n = 367,412) used ≥5 drugs (polypharmacy), 8% (n = 68,100) used ≥10 drugs (excessive polypharmacy) (Table 1). Of the institutionalized individuals (n = 70,500), 68% had polypharmacy, and 19% had excessive polypharmacy. The mean age was 82.3 years (standard deviation (SD) 5.5) for the total population, 82.9 (SD 5.5) for people with polypharmacy, 83.2 (SD 5.5) for people with excessive polypharmacy, and 87.2 years (SD 5.9) for people residing in institution. The institutionalized older adults used more drugs (Median: 6, interquartile range (IQR): 4–9) than the total study population (median: 4, IQR: 2–7).

**Table 1:** Characteristics of the population stratified by polypharmacy, excessive polypharmacy and living arrangement. Aged ≥75 years Sweden, 2013.

	Total population		Polypharmacy, ≥5 drugs5 drugs		Excessive Polypharmacy, ≥10 drugs≥10 drugs		Institution	
	(n = 822,619)		(n = 367,412)		(n = 68,100)		(n = 70,500)	
	%	n	%	n	%	n	%	n
Age								
75–84	68.9	566,527	63.3	232,418	61.2	41,648	32.8	23,006
85–94	28.9	237,899	34.2	125,566	36.3	24,701	56.6	39,653
95+	2.2	18,193	2.6	9,428	2.6	1,751	10.6	7,391
Women	58.9	484,498	61.3	225,106	64.8	44,096	71.4	50,028
Institution	8.5	70,050	13.1	47,989	19.0	12,946	100	70,050
Number of drugs								
0	13.3	109,498	0.0	0	0.0	0	4.8	3,364
1–4	42.0	345,709	0.0	0	0.0	0	26.7	18,697
5–9	36.4	299,312	81.5	299,312	0.0	0	50.0	35,043
≥10	8.3	68,100	18.5	68,100	100	68,100	18.5	12,946
Number of drugs, median (IQR)	4	2 to 7	7	6 to 9	11	10 to 13	6	4 to 9

The 10 most frequently used drug classes (ATC 3^rd^ level) and drug compounds (ATC 5^th^ level) among people with polypharmacy, excessive polypharmacy, and institutionalized individuals are presented in Table 2. Cardiovascular drugs, analgesics, and psychotropic drugs dominated the lists in all three groups (i.e. people with polypharmacy, excessive polypharmacy and institutionalized). Drug classes such as antithrombotic drugs (ATC code B01A) and minor analgesics (N02B) were among the five most commonly used drug classes in all three groups, and consequently acetylsalicylic acid (B01AC06) and paracetamol (N02BE01) were the two most frequently used drug compounds. Lipid modifying drugs (C10A)/simvastatin (C10AA01), and ACE inhibitors (C09A)/enalapril (C09AA02) were among the 10 most frequently used drug classes/compounds among people with polypharmacy, but not in the institutionalized population. Antidepressants (N06A)/citalopram (N06AB04), anxiolytics (N05B)/oxazepam (N05BA04) were more frequently used among institutionalized than community-dwelling older people. The most frequently used drug classes and compounds for persons aged ≥65 years (n = 1,051,115) are available as supporting information, S1 Table.

**Table 2:** The 10 most commonly used drug classes (3^rd^ level ATC) and drug compounds (5^th^ level ATC code) among older adults with polypharmacy, excessive polypharmacy and living in institutions. Aged ≥75 years, Sweden, 2013.

	Polypharmacy (n = 376,412)			Excessive polypharmacy (68,100)				Institution (n = 70,500)			
**ATC, 3**^**rd**^ **level**	**Drug class**	**%**	**n**	**ATC, 3rd level**	**Drug classes**	**%**	**n**	**ATC, 3rd level**	**Drug classes**	**%**	**n**
B01A	Antithrombotic agents	61.1	224,412	B01A	Antithrombotic agents	71.6	48,726	N02B	Minor analgesics	48.0	33,648
C07A	Beta-blockers	51.9	190,696	C07A	Beta-blockers	62.2	42,359	B01A	Antithrombotic agents	44.3	31,062
C10A	Lipid modifying agents	38.5	141,435	N02B	Minor analgesics	54.6	37,176	N06A	Antidepressants	42.2	29,563
N02B	Minor analgesics	32.3	118,701	A02B	Drug for peptic ulcer	49.3	33,576	B03B	Vitamin B12, folic acid	32.7	22,934
B03B	Vitamin B12, folic acid	30.1	110,429	C03C	High-ceiling diuretics	48.6	33,085	C03C	High-ceiling diuretics	30.4	21,273
C08C	Selective calcium channel blockers	28.8	105,799	B03B	Vitamin B12, folic acid	45.8	31,174	C07A	Beta-blockers	30.3	21,192
C03C	High-ceiling diuretics	28.1	103,294	C10A	Lipid modifying agents	45.8	31,160	N05C	Hypnotics/sedatives	26.4	18,510
A02B	Drug for peptic ulcer	27.8	102,062	N05C	Hypnotics/sedatives	44.4	30,261	A02B	Drug for peptic ulcer	26.3	18,417
N05C	Hypnotics/sedatives	27.6	101,303	N06A	Antidepressants	37.7	25,660	N05B	Anxiolytics	23.5	16,463
C09A	ACE inhibitors	25.4	93,477	C08C	Selective calcium channel blockers	32.6	22,169	A06A	Laxatives	19.6	13,729
**ATC, 5**^**th**^ **level**	**Drug compound**	**%**	**n**	**ATC, 5**^**th**^ **level**	**Drug compound**	**%**	**n**	**ATC, 5**^**th**^ **level**	**Drug compound**	**%**	**n**
B01AC06	Acetylsalicylic acid	44.3	162,727	N02BE01	Paracetamol	54.6	37,160	N02BE01	Paracetamol	48.0	33,636
N02BE01	Paracetamol	32.3	118,614	B01AC06	Acetylsalicylic acid	51.4	34,966	B01AC06	Acetylsalicylic acid	36.4	25,521
C07AB02	Metoprolol	30.9	113,642	C03CA01	Furosemide	48.1	32,781	C03CA01	Furosemide	30.2	21,146
C10AA01	Simvastatin	29.3	107,580	A02BC01	Omeprazole	41.3	28,130	B03BA01	Cyanocobalamin	28.4	19,873
C03CA01	Furosemide	27.9	102,522	B03BA01	Cyanocobalamin	39.4	26,848	A02BC01	Omeprazole	23.4	16,380
B03BA01	Cyanocobalamin	25.9	95,275	C07AB02	Metoprolol	36.1	24,614	N06AB04	Citalopram	21.5	15,084
A02BC01	Omeprazole	23.5	86,211	C10AA01	Simvastatin	33.8	23,008	N05BA04	Oxazepam	20.0	13,976
C09AA02	Enalapril	18.1	66,546	N05CF01	Zopiclone	27.5	18,744	C07AB02	Metoprolol	18.3	12,797
N05CF01	Zopiclone	16.6	60,792	A12AX	Calcium + vitamin D combinations	26.1	17,767	N05CF01	Zopiclone	18.5	12,975
A12AX	Calcium + vitamin D	16.4	60,137	H03AA01	Levothyroxine sodium	21.3	14,515	B03BB01	Folic acid	16.4	11,460

The reduction in the prevalence of polypharmacy by excluding drugs among the top 10 drug compounds (Panel A) and classes (Panel B) is depicted in Fig 1. The prevalence of polypharmacy was reduced from 45% to 36% when excluding the three most frequently used drug compounds; to 31% when excluding the top 5 drug compounds and to 22% when excluding all the top 10 drug compounds (Panel A). In total, this represents a 51% reduction of the prevalence of polypharmacy. For the drug classes (Panel B), the prevalence of polypharmacy was reduced from 45% to 31% when excluding the three most frequently used drug classes, to 25% when excluding the top 5 drug classes and to 14% when excluding all the top 10 drug classes. In total, the prevalence of polypharmacy was reduced by 69% when removing the ten most frequently used drug classes.

In Fig 2, the 10 most frequently used drug classes (Panel A) and drug compounds (Panel B) are presented according to the concurrent number of other drugs used. Only 1% to 3% of the users of one of the 10 most frequently drug classes used that drug class as monotherapy and the corresponding proportion for drug compounds was also 1% to 3% in the total study population (n = 822,619). The majority of the users of either one of the 10 most frequent drugs used at least four other drugs classes (68%-85%) or drug compounds (66%-85%) in combination with that specific drug.

## Discussion

In this large unselected nationwide study of Swedes aged 75 years and older, the prevalence of polypharmacy (≥5 drugs) was 45% and the prevalence of excessive polypharmacy (≥10 drugs) was 8%. The use of drugs was centered on cardiovascular drugs, analgesics and psychotropic drugs among people with polypharmacy, excessive polypharmacy and people residing in an institution respectively. A handful of drugs make a large contribution to the overall prevalence of polypharmacy. The prevalence of polypharmacy would be halved by excluding the ten most frequently used drug compounds.

The most frequently used drugs among people aged 75 years with polypharmacy, excessive polypharmacy, and institutionalized were fairly similar and mainly included cardiovascular drugs, analgesics and psychotropic drugs (the most frequent drugs was also similar among persons aged ≥65 years with polypharmacy). This is in line with previous studies investigating drug use among very old adults [4,13,14]. Institutionalized individuals used some cardiovascular drug classes less frequently, such as lipid modifying agents and ACE inhibitors. This may reflect that people in institutions are on average older, and these drug have been reported to be used at lower rates among very old people [13]. However, this might also reflect that there are different prescribing patterns in institutions relative to the community setting [4,17]. People residing in institutions in Sweden are frail and often have cognitive impairment and limited remaining life expectancy [26,27]. This could contribute to the lower use of preventive treatments (e.g., lipid lowering drugs) in these settings [28]. Contrary, antidepressants and anxiolytics were more common in institutions than among community-dwellers [4,13]. In this study, 42% of the institutionalized individuals used an antidepressant.

The use of drugs was concentrated around some frequently prescribed drugs. The prevalence of polypharmacy would be halved, from 45% to 23% if the six most frequently used drug classes were removed. This suggests that drug use among older adults is concentrated around a limited number of drug classes. Several attempts to reduce polypharmacy in clinical settings have been made [18–22]. Eliminating the ten most frequently used drugs is unrealistic because many of them are essential drug treatments with well-known safety profiles. However, these drugs represent the drugs for which the largest gains in lowering the prevalence of polypharmacy could be accomplished if eliminated. Our results points to that reducing polypharmacy at the population level would be a large undertaking because even in the simplistic example of excluding the 10 most used drug compounds, the prevalence would only be halved. If polypharmacy *per se* should be targeted, our findings highlight that providing person-centered and effective deprescribing for the large group of older adults with polypharmacy will indeed be a challenge.

The majority of individuals using one of the 10 most frequently used drugs were concurrently using at least four other drugs used drugs (66–85%). This highlights that real-life drug use among older adults is indeed different from the typical clinical drug trial that tests one drug for one disease at the time [9–11].

### Strength and limitations

The main strength of this study is the large and unselected nationwide study population. We provide nationally representative figures on the concurrent use of drugs with detailed data on all the prescribed and dispensed drugs. Furthermore, the registers also hold information on the persons’ current living arrangement (community vs. institution). It has earlier been shown that living arrangement is an essential factor in drug utilization studies of older people [4].

There are also some limitations to this study. The SPDR does not include over-the-counter drugs, drugs used in hospitals and drugs provided from nursing home supply rooms. Thus, we will most likely underestimate the prevalence of polypharmacy. In addition, we only count combined products (including more than one active ingredient) as one drug, this will also lead to an underestimation of the number of active ingredients used. Moreover, we only know that the prescriptions have been filled at the pharmacy, but we do not know to what extent the patient adheres to the treatment. This will most likely lead to an overestimation of the prevalence of polypharmacy. Furthermore, the analysis of how the prevalence of polypharmacy changes when excluding the ten most commonly used drugs is a simulation test that is unrealistic in the clinical setting. However, with this, rather than suggesting that the most frequently used drugs should be removed, we wished to shed a light on to what extent polypharmacy is concentrated around the use of certain drugs.

In sum, almost half of the population aged ≥75 years used five or more drugs concurrently (polypharmacy). The majority of the most common drugs are used concurrently with at least four other drugs. This highlights the complexity of older peoples’ drug therapy. We also show that, in Sweden, removing as much as the ten most commonly used drug compounds would only reduce the prevalence of polypharmacy by half. This suggests that reducing the prevalence of polypharmacy on a population level will indeed be a challenge because providing person-centered and safe deprescribing for almost half of the population is a major undertaking.

## Supporting information

S1 Table The 10 most commonly used drug classes (3rd level ATC) and drug compounds (5th level ATC code) for people using ≥5 drugs, ≥10 drugs and institutionalized. Aged 65–74 years (n = 1,051,115), Sweden 2013.
(DOCX)
