# Supplementary material for: The composition of polypharmacy: A register-based study of Swedes aged 75 years and older
Source: PLoS One. 2018 Mar 29;13(3):e0194892. doi: 10.1371/journal.pone.0194892 (PMC5875802; doi:10.1371/journal.pone.0194892)
Supplement: S1 Table — Aged 65–74 years (n = 1,051,115), Sweden 2013. (DOCX) [file pone.0194892.s001.docx]

**S1 Table.** **The 10 most commonly used drug classes (3rd level ATC) and drug compounds (5th level ATC code) for people using ≥5 drugs, ≥10 drugs and institutionalized. Aged 65-74 years (n=1,051,115), Sweden 2013**

|  | Polypharmacy (n= 214,904) | | |  | Excessive polypharmacy (39,243) | | | |  | Institution (n=7,691) | | | |
| --- | --- | --- | --- | --- | --- | --- | --- | --- | --- | --- | --- | --- | --- |
| **ATC, 3^rd^ level** | **Drug class** | **%** | **n** |  | **ATC, 3rd level** | **Drug classes** | **%** | **n** |  | **ATC, 3rd level** | **Drug classes** | **%** | **n** |
| C10A | Lipid modifying agents | 51.3 | 110,271 |  | B01A | Antithrombotic agents | 62.8 | 24,645 |  | N06A | Antidepressants | 44.3 | 3,407 |
| B01A | Antithrombotic agents | 49.9 | 107,120 |  | C10A | Lipid modifying agents | 61.4 | 24,091 |  | N02B | Minor analgesics | 36.0 | 2,768 |
| C07A | Beta-blockers | 48.3 | 103,824 |  | C07A | Beta-blockers | 59.4 | 23,300 |  | B01A | Antithrombotic agents | 33.9 | 2,605 |
| C08C | Selective calcium channel blockers | 31.3 | 67,313 |  | A02B | Drug for peptic ulcer | 51.2 | 20,099 |  | N05C | Hypnotics/sedatives | 28.2 | 2,167 |
| C09A | ACE inhibitors | 25.5 | 54,707 |  | N02B | Minor analgesics | 43.8 | 17,183 |  | A02B | Drug for peptic ulcer | 25.3 | 1,948 |
| A02B | Drug for peptic ulcer | 24.2 | 52,069 |  | N05C | Hypnotics/sedatives | 38.6 | 15,152 |  | B03B | Vitamin B12, folic acid | 25.3 | 1,948 |
| N05C | Hypnotics/sedatives | 18.6 | 39,856 |  | C08C | Selective calcium channel blockers | 36.2 | 14,200 |  | C07A | Beta-blockers | 23.3 | 1,795 |
| N02B | Minor analgesics | 17.9 | 38,419 |  | N06A | Antidepressants | 34.0 | 13,334 |  | C10A | Lipid modifying agents | 20.8 | 1,603 |
| N06A | Antidepressants | 16.6 | 35,618 |  | B03B | Vitamin B12, folic acid | 34.0 | 13,328 |  | C03C | High-ceiling diuretics | 17.8 | 1,366 |
| B03B | Vitamin B12, folic acid | 15.8 | 33,890 |  | C03C | High-ceiling diuretics | 33.2 | 13,027 |  | C09A | ACE inhibitors | 15.9 | 1,221 |
| **ATC, 5^th^ level** | **Drug compound** | **%** | **n** |  | **ATC, 5^th^ level** | **Drug compound** | **%** | **n** |  | **ATC, 5^th^ level** | **Drug compound** | **%** | **n** |
| B01AC06 | Acetylsalicylic acid | 37.6 | 80,811 |  | B01AC06 | Acetylsalicylic acid | 46.4 | 18,198 |  | N02BE01 | Paracetamol | 36.0 | 2,768 |
| C10AA01 | Simvastatin | 34.7 | 74,628 |  | N02BE01 | Paracetamol | 43.8 | 17,175 |  | B01AC06 | Acetylsalicylic acid | 26.7 | 2,053 |
| C07AB02 | Metoprolol | 28.6 | 61,483 |  | A02BC01 | Omeprazole | 40.6 | 15,948 |  | A02BC01 | Omeprazole | 21.4 | 1,644 |
| A02BC01 | Omeprazole | 19.8 | 42,484 |  | C10AA01 | Simvastatin | 38.9 | 15,251 |  | B03BA01 | Cyanocobalamin | 19.5 | 1,502 |
| C08CA01 | Amlodipine | 18.5 | 39,831 |  | C07AB02 | Metoprolol | 33.3 | 13,065 |  | C10AA01 | Simvastatin | 17.7 | 1,362 |
| C09AA02 | Enalapril | 18.3 | 39,320 |  | C03CA01 | Furosemide | 32.8 | 12,860 |  | C03CA01 | Furosemide | 17.6 | 1,356 |
| N02BE01 | Paracetamol | 17.9 | 38,367 |  | B03BA01 | Cyanocobalamin | 26.9 | 10,539 |  | N05CF01 | Zopiclone | 17.2 | 1,326 |
| B03BA01 | Cyanocobalamin | 12.7 | 27,281 |  | C08CA01 | Amlodipine | 21.0 | 8,235 |  | C07AB02 | Metoprolol | 14.3 | 1,103 |
| H03AA01 | Levothyroxine sodium | 11.9 | 25,575 |  | A12AX | Calcium + vitamin D combinations | 20.5 | 8,062 |  | C09AA02 | Enalapril | 12.2 | 936 |
| C03CA01 | Furosemide | 10.6 | 22,852 |  | N05CF01 | Zopiclone | 20.3 | 7,968 |  | H03AA01 | Levothyroxine sodium | 9.5 | 728 |
